# Evaluating the Heart Wise Exercise™ program: a model for safe community exercise programming

**DOI:** 10.1186/s12889-016-2866-7

**Published:** 2016-02-27

**Authors:** Jennifer L. Reed, Jennifer M. Harris, Liz Midence, Elizabeth B. Yee, Sherry L. Grace

**Affiliations:** Division of Prevention and Rehabilitation, University of Ottawa Heart Institute, 40 Ruskin Street, Ottawa, Ontario K1Y 4W7 Canada; School of Kinesiology and Health Science, York University, Toronto, Ontario Canada; Toronto Rehabilitation Institute, University Health Network, Toronto, Ontario Canada

**Keywords:** Community exercise, Cardiovascular rehabilitation, Exercise training

## Abstract

**Background:**

Greater availability of community exercise facilities is recommended to promote physical activity in the large number of people with chronic disease. The Heart Wise Exercise (HWE) program encourages existing community-based exercise facilities to build capacity to serve such patients, by working with interested facilities to ensure they meet safety criteria, and educating exercise leaders.

**Methods:**

Using a piloted checklist, 45 HWE programs were audited for the six HWE criteria (outlined below) in the greater Ottawa and Toronto areas of Ontario, Canada. A survey was also administered to a convenience sample of HWE program participants (*N* = 127).

**Results:**

Criteria 1: 71 % of leaders encouraged daily aerobic exercise; participants reported engaging in 194 min/week of aerobic exercise. Criteria 2: 100 % of programs incorporated a warm-up and cool-down, and 84 % encouraged self-monitoring during class. Criteria 3: 98 % of programs offered options for participants to exercise at their appropriate intensity. Criteria 4: HWE participants reported having chronic conditions including arthritis (41 %), osteoporosis (26 %) diabetes (8 %), heart disease (6 %) and chronic obstructive pulmonary disease (6 %). Criteria 5: 93 % of instructors offered health screening for participants. Criteria 6: 100 % of sites had automated external defibrillators, and 90 % of the instructors were aware of the documented emergency plan. The exercise leaders reported being ‘knowledgeable/comfortable/confident’ in providing exercise guidance to, and having clients with chronic health conditions; directing clients to other services; offering exercise intensity options; helping clients listen to their bodies; and, encouraging clients to provide information regarding their health. The participants reported being, on average, ‘somewhat happy’ to ‘very happy’ with HWE locations; program dates and times; leaders’ knowledge of disease and exercise; exercise intensity; cost; and, social aspect.

**Conclusions:**

HWE programs are safe and appropriate for persons with varying chronic health conditions, and participants are satisfied with and will likely continue attending their HWE classes. Future efforts should be directed at increasing awareness of HWE programs among referring healthcare professionals and participants at risk of chronic conditions. The HWE training program should emphasize that HWE leaders regularly encourage self-monitoring and daily aerobic exercise, which is well-known to reduce the burden of many chronic diseases.

**Electronic supplementary material:**

The online version of this article (doi:10.1186/s12889-016-2866-7) contains supplementary material, which is available to authorized users.

## Background

Cardiovascular diseases are among the leading causes of morbidity worldwide [1]. Persons with cardiovascular disease require long-term management if recurrent events and other complications are to be postponed or prevented. Cardiovascular outcomes are largely dependent on gains in cardiometabolic fitness [2, 3]. Daily exercise is a fundamental and modifiable contributor to cardiometabolic fitness. Guidelines recommend patients accumulate 30 to 60 min of moderate-to-vigorous intensity aerobic exercise on most, but preferably all, days of the week for secondary prevention [4].

Although cardiovascular rehabilitation programs are successful in ensuring patients initiate and engage in exercise, long-term maintenance of exercise remains a challenge [5–9]. Among patients who complete cardiovascular rehabilitation, less than 50 % continue to meet exercise recommendations thereafter [10]. Safe and appropriate facilities are therefore needed to promote exercise maintenance [11, 12]. However, there is insufficient capacity in existing cardiovascular rehabilitation programs for graduated patients to continue exercise, as many programs cannot even meet the demand for new patients. While some cardiovascular rehabilitation programs offer Phase IV, or maintenance programming, at a cost to patients, these programs may be unaffordable, inconveniently located in terms of travel time and distance, and the limited class dates and times available for maintenance programming may be inconvenient for patients. Moreover, there is scant research addressing degree of use of Phase IV rehabilitation, and therefore it is not known whether there are sufficient services and whether they are meeting patient needs.

Availability of low-cost and accessible exercise facilities in the community therefore represents a more feasible approach to encouraging continued exercise in cardiac rehabilitation graduates. Indeed, in the World Health Organization’s Global Action Plan on the Prevention and Control of Non-Communicable Diseases 2013–2020, they specifically recommend “increased availability of safe environments in […] recreational spaces to encourage physical activity” (p. 51) [13]. However, previous research has shown that cardiovascular rehabilitation graduates perceived instructors in the community as having limited knowledge and skills in relation to supervision of exercise in people with their condition, and they can feel like an “outsider” in relation to young, healthy attendees [14]. Thus, there is a need to broadly offer safe, accessible exercise environments for patients with cardiovascular, among other, chronic diseases.

The Heart Wise Exercise™ model (heartwise.ottawaheart.ca; HWE) was consequently developed. HWE developers identified and encouraged existing community-based exercise programs to build capacity for serving patients with chronic disease, with the goal of broad delivery of safe and appropriate exercise classes for stable outpatients with chronic disease. This is achieved by working with interested facilities to ensure they meet specific safety criteria, educating their exercise leaders, and then providing HWE materials to these facilities to market their Heart Wise Exercise sessions to clients. The six specific criteria programs must meet to be recognized as “Heart Wise” are: (1) encouraging daily aerobic exercise; (2) incorporating a warm-up, cool down and self-monitoring; (3) allowing participants to exercise at a safe level and offering options to modify intensity; (4) accepting participants with chronic health conditions; (5) offering health screening for all participants; and, (6) having a documented emergency plan that is known to all fitness leaders (who have current cardiopulmonary resuscitation certification), phone access to local paramedic services and having an automated external defibrillator (AED) on-site.

Models similar to HWE have been developed in other jurisdictions [15, 16]. However, there is a dearth of formal evaluation of these models from the lens of the exercise facility and the participants. Therefore, the objectives of this study were to evaluate: (1) whether HWE programs were meeting the six criteria for safety; (2) whether HWE programs are being used by persons with chronic health conditions as intended; and, (3) the perceptions and experiences of these persons with the HWE program.

## Methods

This was a cross-sectional study using mixed-methods. Audits of HWE programs and survey results from HWE program participants are presented. The study protocol was approved by York University’s Office of Research Ethics (e2014 - 376). The Ottawa Health Sciences Network Research Ethics Board granted an ethics approval exemption for this study.

### Setting

The HWE program was launched in 2007 in the greater Ottawa area of Ontario, Canada. Community-based exercise facilities, both private and publically-funded, were approached regarding the HWE model. Facilities offering both group and individual exercise sessions (e.g., personal training) are eligible to join HWE. Steps required before a formal HWE agreement was signed included a site visit to ensure the six criteria were met, completion of a HWE training program by the fitness leader(s), and agreement to the Terms and Conditions, which include a statement that the program agrees to be audited for quality purposes.

The HWE program was expanded to other regions in the province of Ontario. In particular, it was also expanded to the greater Toronto area in 2009. Facilities put HWE signs in their reception areas and designate specific classes as HWE.

### Procedures

Auditors were university-level kinesiology students who received HWE program training, and were instructed on the elements of the audit form and its’ application in the field. The auditors sent a standardized email to facility contacts to arrange a site audit of HWE classes (i.e., HWE logo, advertised for clients’ chronic disease). If more than one HWE class was offered per week at a particular site, the auditors evaluated a subsequent class, if the site and exercise leaders were agreeable.

On the day of the audit, the auditors first recorded observations from the reception area of the facility, before introducing themselves to the exercise leaders. Exercise leaders were often not aware the audit would be occurring. The audit evaluated whether the HWE-designated session met program criteria using a standardized checklist (see Additional file 1). Some of the audit questions were asked of the fitness leaders prior to the beginning of the class (see notation in Additional file 1). HWE classes were then observed, as well as post-program interactions between participants and exercise leaders.

A survey was administered to a convenience sample of HWE program participants at the end of the audited classes. Participants were provided three options for completing the survey. They could (1) complete a paper-based format at the end of the class, (2) take the paper-based survey home to complete (they were provided with a pre-paid, self-addressed envelope), or (3) complete the survey online at home (SurveyMonkey, Palo Alto, California, USA). Participants choosing the latter option were provided a sheet with a link to the survey to take home. All audits were completed between October, 2014 and March, 2015.

### Sample: sites and participants

At the time of the study, there were 230 facilities formally designated as HWE across Ontario, of which 47 (20.4 %) were in Toronto and 51 (22.2 %) were in Ottawa. A convenience sample of HWE programs offering group exercise classes in the Toronto and Ottawa areas were approached. The facilities were selected if they had signed on to the HWE program at least two months prior, were not a pilot site for the audit checklist, and they had not been informally audited in the last six months. Individual conditioning sessions were excluded.

A convenience sample of HWE class participants were approached to participate in the survey. Auditors attempted to invite all exercise session participants to complete the survey by asking the exercise leader to announce the survey at the end of all audited classes, however this was not possible in all cases (e.g., time didn’t allow, exercise leader declined or forgot, some participants left before the end of the class). Inclusion criteria were being able to read and understand English.

### Measures

The standardized checklist for the audits (see Additional file 1) was based on the six HWE criteria. The audit form was piloted in October 2014 at several sites in the greater Ottawa area. Both the audit form and participant survey, as described below, were developed by experts in the areas of community programming, exercise training, chronic disease management, and program evaluation.

The participant survey items were investigator-generated. Questions pertained to chronic disease diagnoses and risk factors, how they became aware of the HWE program, their satisfaction with the exercise class, their frequency and duration of participation, as well as some items related to HWE criteria. Response options were primarily multiple-choice or Likert-type scales. Again, the items were piloted at several sites in the greater Ottawa area.

### Statistical analyses

A descriptive examination of audit findings and participant survey responses was performed. Data are reported as means ± standard deviations, unless otherwise noted. Valid percentages were reported when there was missing data (i.e., the denominator was reduced to reflect not the total sample size but the number of valid responses). All data were analyzed using IBM SPSS for Windows (version 23; Chicago, IL, USA).

## Results

### Exercise facility and leader characteristics

**Fig. 1:**
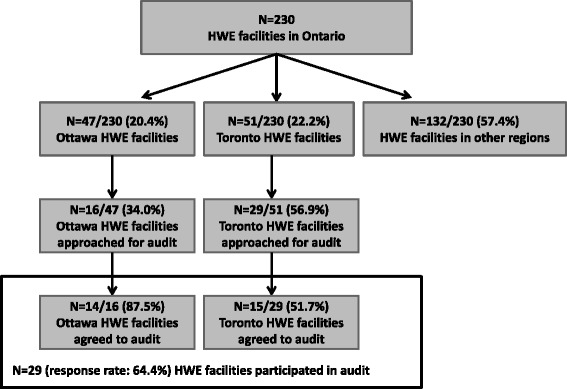
Flow chart of HWE facilities, classes and audits

In Toronto, 15 of 29 (51.7 %) HWE facilities approached agreed to an audit, and in Ottawa 14 of 16 (87.5 %) HWE facilities agreed (*N* = 29 facilities audited overall; 64.4 % response rate; Fig. 1). Six (20.7 %) of these facilities were privately-funded. At fourteen (48.3 %) facilities, two or more classes were audited, for a total of 45 audits. Audited classes included aquafit, yoga, aerobics, cycling, chair exercise classes, Zumba, resistance classes, as well as core strengthening sessions.

At 24 (57.1 %) audits, HWE promotional materials were visibly posted. When the front desks clerks were asked whether there are HWE classes at their facility, 32 (76.2 %) were aware of such classes. Twenty-six (61.9 %) front desk clerks had basic knowledge of the HWE program.

**Fig. 2:**
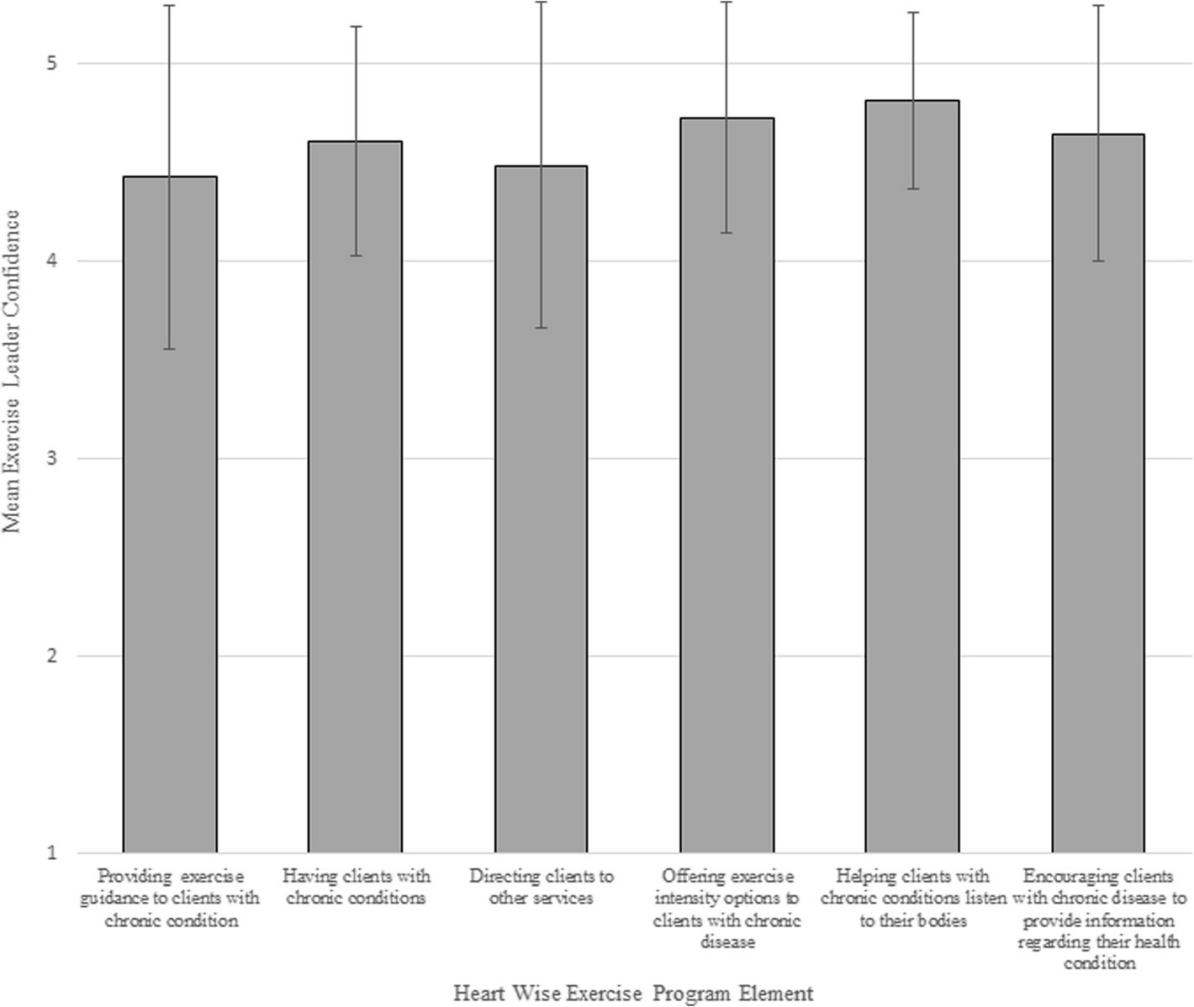
Mean Exercise Leader confidence with Heart Wise Exercise program elements. Response options ranged from 1 “not at all knowledgeable/comfortable/confident” to 5 “extremely knowledgeable/comfortable/confident”. Error bars denote standard deviation

All of the exercise leaders had formal recreation, fitness and/or kinesiology training (*n* = 45, 100.0 %). This was most often CanFit Pro (*n* = 23, 52.3 %), followed by YMCA certification (*n* = 12, 27.3 %). As shown in Fig. 2, the leaders’ confidence in relation to the HWE principles was very high. Specifically, the mean score for helping clients with chronic conditions listen to their bodies was 4.8 ± 0.4; encouraging clients with chronic disease to provide information regarding their health condition was 4.7 ± 0.7; offering exercise intensity options to clients with chronic disease was 4.7 ± 0.6; having clients with chronic conditions was 4.6 ± 0.6; directing clients to other services was 4.5 ± 0.8; and providing exercise guidance to clients with chronic conditions was 4.4 ± 0.9. Two (4.5 %) fitness leaders reported there had ever been a cardiac-related incident in their HWE class. When asked if they would like further training to develop their skills in leading HWE classes, 32 (82.1 %) responded affirmatively.

### HWE class audits

Results of the audit in relation to the six HWE criteria are shown in Table 1. As shown, fidelity to the criteria was very high. Observations of HWE classes revealed that 44 (97.8 %) fitness leaders were able to identify the signs of over-exertion in their class participants. In 42 (97.7 %) classes, auditors observed that exercise options were provided to participants so they could choose an appropriate level of exercise intensity. Participants had the opportunity to interact and ask questions of the fitness leader after 38 (86.4 %) classes.

**Table 1:** Results of HWE audits, *N* = 45

Criteria	n (%) meeting
1. Encouraging daily aerobic exercise	32/45 (71.1 %)
2a. Incorporating a warm-up	44/44 (100.0 %)
2b. Incorporated a cool down	42/42 (100.0 %)
2c. Encouraging self-monitoring	38/45 (84.4 %)
3. Allowing participants to exercise at a safe level and offering options to modify intensity	42/43 (97.7 %)
4. Accepting participants with chronic health conditions^a^	29/29 (100.0 %)
5. Offering health screening for all participants^a^	27/29 (93.1 %)
6a. Having a documented emergency plan that is known to all fitness leaders^a^	26/29 (89.7 %)
6b. Fitness leaders have current cardiopulmonary resuscitation certification^a^	27/29 (93.1 %)
6c. Phone access to local paramedic services^a^	28/29 (96.6 %)
6d. Having an automated external defibrillator on-site^a^	28/28 (100.0 %)

^a^assessed at the facility-level. All other items assessed at the exercise class-level. Please note items 6a and b were assessed at both the facility and exercise class levels

### HWE participants

A total of 127 HWE participants from 18 (40.0 %) audited locations completed the survey online (49.6 %) or on paper (50.4 %) at the end of class. Their characteristics are shown in Table 2. On average, the participants were primarily middle-aged women, who were highly physically active. Their most common chronic health diagnoses were arthritis and osteoporosis. Their most preponderant risk factors overweight and hypertension. As shown, very few were cardiac rehabilitation graduates.

**Table 2:** Characteristics of HWE participants, *N* = 127

Characteristic	n (%)/ mean ± SD
Age	59.7 ± 15.9
Sex (% female)	108 (89.3 %)
Toronto Area resident	65 (51.2 %)
Health conditions	
Arthritis	52 (41.3 %)
Osteoporosis	32 (25.8 %)
Diabetes	10 (7.9 %)
Cardiac disease	7 (5.6 %)
Chronic obstructive pulmonary disease	7 (5.6 %)
Mental condition	6 (4.8 %)
Cerebral event (TIA/stroke)	6 (4.8 %)
Cardiac intervention (PCI or CABG surgery)	4 (3.2 %)
Myocardial infarction	1 (0.8 %)
Risk factors	
Overweight	40 (32.3 %)
Hypertension	34 (26.8 %)
Hyperlipidemia	23 (18.4 %)
Sought emergency medical care in past year for chronic disease	7 (5.6 %)
Pre-diabetes	7 (5.6 %)
Previous participation in cardiovascular rehabilitation	5 (4.1 %)
Smoker	2 (1.7 %)
Minutes of moderate or vigorous intensity exercise per week	194.1 ± 190.7

*SD* standard deviation, *HWE* Heart Wise Exercise, *TIA* transient ischemic attack, *PCI* percutaneous coronary intervention, *CABG* coronary artery bypass graft

Most participants had learned of the HWE program through a brochure (*n* = 58, 46.0 %), followed by exercise leaders (*n* = 26, 20.6 %) and friends (*n* = 11, 8.7 %). Twenty (16.4 %) participants were aware their class was designated as HWE and that is the reason why they joined, whereas 41 (33.6 %) participants knew but this was not the reason why they joined, and 44 (36.1 %) were not aware it was a HWE class.

When asked how long they had been coming to the HWE class, three (2.5 %) reported this was their first time, seven (5.7 %) reported a few weeks, nine (7.4 %) reported a month or two, and 103 (84.4 %) reported a few months or more. When asked whether they intend to continue participating in HWE classes on a scale of 1 ‘not at all’ to 5 ‘very likely’, the mean score was 4.8 ± 0.5 (standard deviation). One-hundred and six (86.9 %) participants reported that they regularly engage in other exercise in addition to their participation in their HWE class. Participants travelled a mean of 13.2 ± 12.2 (median =10.0) minutes one-way to the HWE class.

**Fig. 3:**
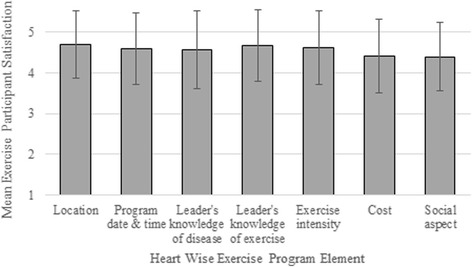
Mean Exercise Participant satisfaction with Heart Wise Exercise program elements. Response options ranged from 1 “very unhappy” to 5 “very happy”. Error bars denote standard deviation

As shown in Fig. 3, the participants’ satisfaction with HWE program elements was very high. The mean scores and standard deviation, in descending order were: location (4.7 ± 0.8); leader’s knowledge of exercise (4.7 ± 0.9); program date and time (4.6 ± 0.9); leader’s knowledge of chronic disease (4.6 ± 1.0); intensity of the exercise (4.6 ± 0.9); cost (4.4 ± 0.9); and, the social aspect (4.4 ± 0.9).

With regard to HWE criteria, when asked whether they were encouraged to “listen to their body” (i.e., exercise intensity self-monitoring) during the class, on a scale of 1 ‘not at all’ to 5 ‘yes, very much’, the mean response was 4.06 ± 1.29. When asked whether their HWE leader invited them to share any health conditions they may have (i.e., health screening), on a scale of 1 ‘never’ to 5 ‘always’, the mean response was 4.13 ± 1.34.

## Discussion

This study was the first to formally evaluate the HWE program, which seeks to identify a wide range of safe and appropriate community programs for those with, or at risk for, cardiovascular or other chronic conditions, and systematically connect patients exiting cardiovascular rehabilitation to nearby facilities for life-long exercise. Findings revealed that HWE-designated exercise sessions were generally delivered in accordance with the six criteria, reached persons with chronic health conditions but not necessarily cardiac rehabilitation graduates, and participants were highly satisfied with HWE sessions.

There is an epidemic of non-communicable diseases globally. Our findings are not only applicable to communities in Ontario, Canada, but also communities worldwide. The World Health Organization's 2015 World Report on Ageing and Health emphasizes that “older people's participation in physical activity is increasingly linked to the environment in which they live” (p.194) and that policy-makers should create environments that promote physical activity [17]. Our evaluation demonstrates that HWE is an effective model to support communities in providing access to safe exercise environments for adults, those with chronic disease and/or those recovering from acute health events.

The two HWE criteria which should be improved upon were encouraging daily aerobic exercise and instructing participants how to self-monitor exercise intensity. Only 71 % of the fitness leaders encouraged daily aerobic exercise. Because the site audits were conducted at a single point in time, it is possible that the fitness leaders frequently encourage daily aerobic exercise, but it was not observed during the audited class specifically. With regard to the latter, there are several practical methods for self-monitoring exercise intensity that are routinely presented in the HWE Training Program for fitness leaders, including heart rate monitoring, the Borg Rating of Perceived Exertion and the Talk Test [18–20]. Audit findings were provided to the facilities, and therefore it is hoped that this feedback will result in improvements in these areas. Providing more or perhaps better examples of how to incorporate these tools into a fitness class and having opportunities to practice delivery of these methods during the workshop may encourage the fitness leaders to use these tools consistently in every session.

HWE programs generally reached highly and consistently active, older females, many of whom had arthritis or osteoporosis. It was surprising how few cardiac rehabilitation graduates were accessing the HWE sessions. In our survey of cardiac rehabilitation graduates in regions where the HWE program is available, almost half of graduates intended to exercise at a community facility post-program [21]. One year post-cardiac rehabilitation, almost two-thirds of graduates reported exercising in the community, of which one-third reported exercising at a HWE facility specifically [21]. However, most respondents reported exercising at home rather than in the community [21]. A similar model has been implemented in Australia following cardiac and pulmonary rehabilitation, and results showed that 60 % attended at least once, and one-third attended at least six sessions (i.e., just over half of those who attended at least once) [16]. In another study of cardiac rehabilitation graduates, many intended to join community-based programs but most discontinued after five or fewer sessions, citing lack of confidence in their ability to exercise without medical supervision and lack of support for their transition as causal factors [14]. As suggested by participants in the latter study, perhaps a better linkage between the community-based program and rehabilitation program should be made prior to program completion.

Awareness and use of the HWE classes could be improved by ensuring greater visibility of session offerings at reception of the community facilities, and on their websites. The HWE developers are currently working to increase awareness of the program, so that it is more highly accessed by chronic disease patients, including those with cardiovascular disease. There is also room for improvement in terms of ensuring staff working at the reception desks of these exercise facilities are aware of the HWE program and what it offers. We also recently partnered with a provincial health and fitness society to offer the HWE fitness leader training to a wider audience.

Our findings show that HWE fitness leaders were confident and knowledgeable instructors in the area of exercise in chronic disease. Recently adapted to allow a more flexible delivery, using a combination of in-person and online modules, the training program has clearly been successful. To date, more than 1,150 fitness leaders have completed the HWE Training Program. Our findings show that HWE fitness leaders are confident and knowledgeable instructors. With the increase in participants with, and at risk for, chronic conditions, having instructors able to meet the needs of these participants, and provide meaningful exercise and social encounters for them will be essential to helping them remain high-functioning and independent. While the exercise leaders were eager to participate in further training, it does appear that the HWE model speaks to some of the suggestions raised by cardiac rehabilitation graduates exercising at non-specific community exercise facilities [14]. In the Training Program, fitness leaders are also informed about chronic disease management programs available for participants who come to their classes but may not have accessed them yet (e.g., cardiovascular rehabilitation, diabetes educators, chronic obstructive pulmonary disease clinics). This ensures participants with chronic disease will know how to exercise safely in the community setting.

Several limitations of the current study must be conceded. Firstly, this was a cross-sectional study with no control or comparison group (e.g., patients not going to HWE or who discontinued attending HWE sessions) and therefore caution is warranted in interpreting or over-generalizing the findings. Second, the current study was conducted using a convenience sample of HWE participants and HWE programs, so the results may have limited generalizability and selection bias may be at play. For example, male participants were under-represented in the participant surveys, and hence the findings are likely less generalizable to male than female exercisers. Third, the HWE contact may have informed the exercise leader of the audits, hence leading to a Hawthorne effect. Replication is warranted in a representative sample using a randomized controlled design before causal conclusions regarding the impact of the HWE model on the delivery of exercise for patients with chronic disease in the community can be drawn. The further limitation pertains to measurement. The audit forms and participant surveys were investigator-generated and not validated. Therefore, there could have been some measurement error. Finally, exercise leaders and exercise session participants self-reported their characteristics and perceptions. Self-report is often biased by recall failure and socially-desirability, leading to an overestimation of physical activity levels.

## Conclusions

This study was the first to evaluate the HWE model which seeks to identify and promote a wide range of safe and appropriate community programs for patients with, or at risk of, chronic disease. HWE programs appear to be safe and appropriate for persons with varying chronic health conditions. The exercise sessions were generally delivered in accordance with the six criteria. The HWE training program should more greatly emphasize that exercise leaders regularly encourage self-monitoring of exercise intensity and daily aerobic exercise among participants. Participants were satisfied with and intend to continue attending their HWE classes. Future efforts should be directed at increasing awareness of HWE offerings among referring health care professionals and participants at risk of, or with, chronic conditions.

## Consent

Written informed consent was obtained from the fitness leaders and participants for publication of this original manuscript. A copy of the written consent is available for review by the Editor of this journal.

## Additional file

Additional file 1:
**Standardized checklist for Heart Wise Exercise-designated sessions.** (PDF 76 kb)

## Abbreviations

AED: automated external defibrillator
HWE: heart wise exercise

## References

[1] World Health Organization. Global status report on non-communicable diseases. World Health Organization. 2014;1–302.

[2] Stone JA, Mancini GMJ, Stone JA, Arthur HM, Suskin NG (2009). The pathophysiology of atherosclerosis and cardiovascular disease. Canadian Guidelines for Cardiac Rehabilitation and Cardiovascular Disease Prevention: Translating Knowledge into Action.

[3] Stone JA, Campbell NR, Genest J, Harris S, Pipe A, Warburton DE, Stone JA, Arthur HM, Suskin NG (2009). Health behaviour interventions and cardiovascular disease risk factor modifications. Canadian Guidelines for Cardiac Rehabilitation and Cardiovascular Disease Prevention.

[4] Canadian Association of Cardiac Rehabilitation (2009). Canadian Guidelines for Cardiac Rehabilitation and Cardiovascular Disease Prevention.

[5] Blanchard CM, Reid RD, Morrin LI, McDonnell L, McGannon K, Rhodes RE (2010). Demographic and clinical determinants of moderate to vigorous physical activity during home-based cardiac rehabilitation: the home-based determinants of exercise (HOME) study. J Cardiopulm Rehabil Prev.

[6] Reid RD, Morrin LI, Pipe AL, Dafoe WA, Higginson LA, Wielgosz AT (2006). Determinants of physical activity after hospitalization for coronary artery disease: the Tracking Exercise After Cardiac Hospitalization (TEACH) Study. Eur J Cardiovasc Prev Rehabil.

[7] Keast ML, Slovinec D'Angelo ME, Nelson CR, Turcotte SE, McDonnell LA, Nadler RE (2013). Randomized trial of Nordic walking in patients with moderate to severe heart failure. Can J Cardiol.

[8] Bock BC, Carmona-Barros RE, Esler JL, Tilkemeier PL (2003). Program participation and physical activity maintenance after cardiac rehabilitation. Behav Modif.

[9] Izawa KP, Yamada S, Oka K, Watanabe S, Omiya K, Iijima S (2004). Long-term exercise maintenance, physical activity, and health-related quality of life after cardiac rehabilitation. Am J Phys Med Rehabil.

[10] Moore SM, Dolansky MA, Ruland CM, Pashkow FJ, Blackburn GG (2003). Predictors of women's exercise maintenance after cardiac rehabilitation. J Cardiopulm Rehabil.

[11] Bentley D, Khan S, Oh P, Grace S, Thomas S (2013). Physical activity behavior 2 to 6 years following cardiac rehabilitation: a socioecological analysis. Clin Cardiol.

[12] Gallant S, Reid RD, Harris J, Grace SL (2015). Location, modality and degree of exercise over 18 months in cardiac rehabilitation graduates. Can J Cardiol.

[13] World Health Organization. Global Action Plan for the Prevention and Control of Noncommunicable diseases. 2013. Geneva, Switzerland.

[14] Clark AM, Mundy C, Catto S, MacIntyre PD (2011). Participation in community-based exercise maintenance programs after completion of hospital-based cardiac rehabilitation: a mixed-method study. J Cardiopulm Rehabil Prev.

[15] Mandic S, Body D, Barclay L, Walker R, Nye ER, Grace SL (2015). Community-Based Cardiac Rehabilitation Maintenance Programs: Use and Effects. Heart Lung Circ.

[16] Adsett J, Hickey A, Nagle A, Mudge A (2013). Implementing a community-based model of exercise training following cardiac, pulmonary, and heart failure rehabilitation. J Cardiopulm Rehabil Prev.

[17] World Health Organization. World Report on Ageing and Health. 2015. Luxembourg, Luxembourg. 1–260

[18] Reed JL, Pipe AL. Practical Approaches to Prescribing Physical Activity and Monitoring Exercise Intensity. Canadian Journal of Cardiology. 2016. (In press). 10.1016/j.cjca.2015.12.024 26897182

[19] Borg G (1998). Borg's Perceived Exertion and Pain Scales.

[20] Reed JL, Pipe AL (2014). The talk test: a useful tool for prescribing and monitoring exercise intensity. Curr Opin Cardiol.

[21] Gallant S, Somanader D, Chessex C, Harris J, Grace SL (2015). Where do patients exercise 1 year post-cardiac rehabilitation?. J Cardiopulm Rehabil Prev.

