# Supplementary material for: Evaluating the Heart Wise Exercise™ program: a model for safe community exercise programming
Source: BMC Public Health. 2016 Feb 27;16:190. doi: 10.1186/s12889-016-2866-7 (PMC4769838; doi:10.1186/s12889-016-2866-7)
Supplement: Additional file 1: — Standardized checklist for Heart Wise Exercise-designated sessions. (PDF 76 kb) [file 12889_2016_2866_MOESM1_ESM.pdf]

HEART WISE EXERCISE
AUDIT FORM 2014/2015

Date: _____ / _____ / 201___

Facility: ____

Class: ____

Type of exercise: ____

Class time: ____

Exercise leader(s): ____

	**Heart Wise Exercise Criteria**	**Are criteria being met?**<br><br>**Yes, No *or* N/A**
Program Design:		
1	The program provides exercise options to help participants choose their appropriate level of intensity.	
2	If there is a competitive nature to the activity, does it enable participants to be exercising at an appropriate level?	
3	Is there time allocated after class for the exercise leader to answer questions of participants?	
Exercise leader(s): SEE ALSO QUESTIONS ON THE NEXT PAGE		
4	Current in CPR and AED use? (verbal yes/no from exercise leader is sufficient)	
5	Encourages participants to do daily physical activity, including outside of the class setting.	
6	Teaches clients how to monitor themselves (i.e., intensity) using RPE and other methods.	
7	Incorporates a gradual and progressive warm-up.	
8	Incorporates a gradual cool down.	
9	Reads screening tools and/or provides verbal screening (e.g., PAR Q, registration/assessment forms or the exercise leader verbally asks the class if anyone has a health condition they would like to share with the leader).<br><br>If not observed, verbally ask the exercise leader if they systematically screen exercise participants for health conditions that could impact their exercise intensity in the class.	
10	Is the exercise leader able to identify the signs of over-exertion?<br>(If the exercise leader does not ask about this during the class, ask them after "What signs and symptoms they watch for in the participants that would suggest the participant was over-exerting themselves?" Enter 'yes' if they are correct.)	

	**Heart Wise Exercise Criteria**	**Are criteria being met?**<br><br>**Yes, No *or* N/A**
11	Ask the exercise leader if there have ever been any cardiac-related incidents in their HWE class?<br><br>If yes, describe here: ________________________________<br>________________________________<br>________________________________	
12a	Ask if the exercise leader would like any further training to further develop their skills in leading HWE classes?	
12b	If yes, please specify: ________________________________	
13a	Ask if the exercise leader has had formal recreation, fitness and/or kinesiology training?	
13b	If yes, what certifications/training do they have? (check all that apply)<br>❑ CSEP<br>❑ Can-Fit Pro<br>❑ ACSM<br>❑ R.Kin<br>❑ OKA<br>❑ YMCA<br>❑ Canadian Centre for Activity and Aging<br>❑ Other, please specify: ____________________	
Safety:		
14	There is an emergency plan that is documented and known to the exercise leader (ask the exercise leader verbally).	
15	Site has phone access to Emergency Medical Services and the exercise leader knows where it is located (verbally ask them where the phone is for EMS).	
16	AED is available, on-site and accessible at all times during Heart Wise Exercise programs.	
Administration:		
17	Heart Wise Exercise promotional material is posted at program/facility.	
18	Ask if the exercise leader has attended a HWE workshop?<br>If no, do they know someone at that centre who has?<br>If yes, have they been taught the HWE principles from them?<br>(If no, please inform the exercise leader about the new on-line modules which will be ready soon, and that they can access them at heartwise.ottawaheart.ca/Toronto/training).	

	**Heart Wise Exercise Criteria**	**Are criteria being met?**<br><br>**Yes, No *or* N/A**
19	Front desk clerks are aware of the program (i.e., ask them "Do you have HWE programs here?").	
20	Front desk clerks have basic knowledge of the Heart Wise Exercise program to answer questions at their specific center. (If they do not know, they should confidently refer you to someone who does. For example, ask them "When are your HWE classes offered?" and/or "What is HWE all about?")	

Please ask the exercise leader:

21. How knowledgeable are you about having clients with chronic conditions in your exercise group?

Not at all knowledgeable	1	2	3	4	5	Extremely knowledgeable

22. How confident are you in your ability to provide appropriate exercise guidance to clients with a chronic condition in your exercise group?

Not at all confident	1	2	3	4	5	Extremely confident

23. How comfortable are you having clients with chronic conditions in your exercise group?

Not at all comfortable	1	2	3	4	5	Extremely comfortable

24. How confident are you in your ability to direct clients to other services as needed? (*diabetes education, cardiac rehabilitation, etc.*)

Not at all confident	1	2	3	4	5	Extremely confident

25. How confident are you in your ability to offer options to your exercise class participants with chronic disease to modify their exercise intensity?

Not at all confident	1	2	3	4	5	Extremely confident

26. How confident are you that you can help your exercise clients with chronic disease to listen to their bodies while exercising in your classes?

Not at all confident	1	2	3	4	5	Extremely confident

27. How comfortable are you in encouraging your chronic disease clients to inform you of their health conditions?

Not at all comfortable	1	2	3	4	5	Extremely comfortable

Comments: ____

____
____
____
____
____
____
____
____
____

HWE auditor's name: ______________________

Student number: ______________________

Signature: ______________________
